# Managing uncertainty in antifungal dosing: antibiograms, therapeutic drug monitoring and drug-drug interactions

**DOI:** 10.1097/QCO.0000000000000740

**Published:** 2021-05-19

**Authors:** Russell E. Lewis, David R. Andes

**Affiliations:** a Department of Medical and Surgical Sciences, University of Bologna. Infectious Diseases, IRCCS S.Orsola-Malpighi University Hospital, Bologna, Italy; b Departments of Medicine and Medical Microbiology & Immunology, University of Wisconsin-Madison, Madison, Wisconsin, USA

**Keywords:** antifungal drug interactions, antifungal pharmacology, antifungal resistance, susceptibility testing, therapeutic drug monitoring

## Abstract

A number of pharmacokinetic and pharmacodynamic factors in critically ill or severely immunosuppressed patients influence the effectiveness of antifungal therapy making dosing less certain. Recent position papers from infectious diseases societies and working groups have proposed methods for dosage individualization of antibiotics in critically ill patients using a combination of population pharmacokinetic models, Monte-Carlo simulation and therapeutic drug monitoring (TDM) to guide dosing. In this review, we examine the current limitations and practical issues of adapting a pharmacometrics-guided dosing approaches to dosing of antifungals in critically ill or severely immunosuppressed populations.

**Recent findings:**

We review the current status of antifungal susceptibility testing and challenges in incorporating TDM into Bayesian dose prediction models. We also discuss issues facing pharmacometrics dosage adjustment of newer targeted chemotherapies that exhibit severe pharmacokinetic drug-drug interactions with triazole antifungals.

**Summary:**

Although knowledge of antifungal pharmacokinetic/pharmacodynamic is maturing, the practical application of these concepts towards point-of-care dosage individualization is still limited. User-friendly pharmacometric models are needed to improve the utility of TDM and management of a growing number of severe pharmacokinetic antifungal drug-drug interactions with targeted chemotherapies.

## INTRODUCTION

The dosing of antifungal agents in severely ill or immunocompromised patients is often uncertain due to changes in antimicrobial pharmacokinetics and pharmacodynamics. Factors influencing drug pharmacokinetics include altered drug bioavailability, changes in the volume of distribution (Vd) and drug penetration at the site of infection, or fluctuating drug clearance associated with the underlying disease or drug interactions [1,2]. The host immune status, disease burden at the time of diagnosis, infection with intrinsically resistant fungal species or development of acquired antifungal resistance during treatment are pharmacodynamic factors can also impact the probability of treatment success.

Pharmacokinetic/pharmacodynamic (PK/PD) studies explore how drug exposures indexed to a standardized measure of drug potency such as the mean inhibitory concentration (MIC) can be used to predict microbiological and clinical effects of antimicrobial treatment [3]. As such, PK/PD analysis is fundamental for predicting which antimicrobial or dosing regimens have the highest probability of treatment success and provides a basis for measuring and minimizing dosing uncertainty.

**FIGURE 1:**
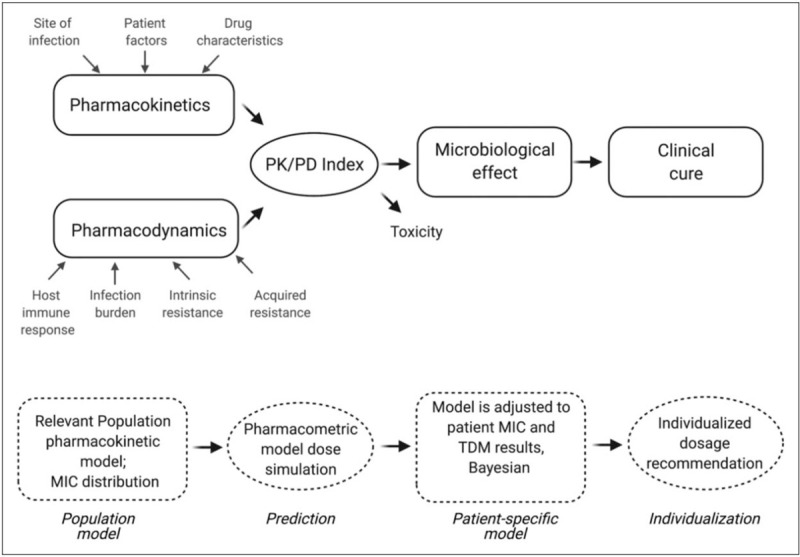
Pharmacological factors contributing to antimicrobial treatment response and the analysis approach (dotted boxes) to dosage individualization.

Several infectious diseases societies and working groups have published position papers on the importance of individualizing the PK/PD performance of antimicrobial therapy in critically ill patients [4,5^▪▪^]. Recommendations have included the use of computerized dosing software with embedded population pharmacokinetic models to predict *a priori* a dosing regimen for an individual patient with a higher probability of PK/PD target attainment for a give range of MICs; *Confirmation* of model-predicted antibiotic exposures through therapeutic drug monitoring (TDM) and patient-specific MIC data, and *a posteriori* personalization of dosing recommendations through Bayesian estimation (Fig. 1). 

**Box 1:**
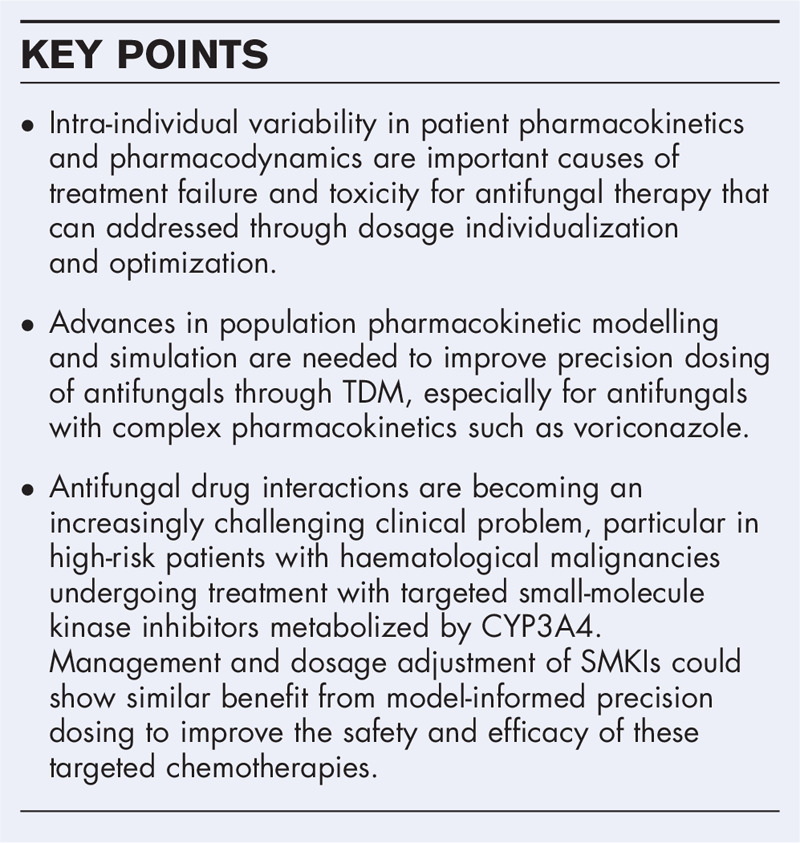
no caption available

Although this approach is becoming more widely applied for antibacterial agents, many challenges remain in adopting this strategy for antifungal dosing. In this review, we examine individualized dosing of antifungal agents at the bedside with a specific focus on antibiograms (MIC testing), TDM and managing drug-drug interactions. We will also explore alternative strategies that can be considered in the absence of definitive microbiology data and individualized PK/PD predictions to reduce antifungal dosing uncertainty.

## PHARMACODYNAMIC VARIABILITY AND THE ANTIBIOGRAM

Antifungal susceptibility testing is the principal tool for identifying pharmacodynamic variability associated with intrinsic or acquired antifungal resistance [6^▪▪^]. The Clinical Laboratory Standards Institute (CLSI) and European Committee on Antimicrobial Susceptibility Testing (EUCAST) have proposed and continually update standardized methods for measuring antifungal MICs *in vitro* against common yeast and moulds [7–9]. Current U.S. and European treatment guidelines for invasive candidiasis recommend susceptibility testing for all *Candida* isolates from blood and deep tissue sites [10,11]. Susceptibility testing for moulds has not been routinely recommended except in regions with high rates of triazole-resistant *Aspergillus* spp. [12,13]. Yet, in a survey of over 4000 acute care hospitals in the U.S. enrolled in the National Healthcare Safety Network, fewer than one in four offered antifungal susceptibility testing onsite or reflexively performed testing in fungal isolates from sterile sources (i.e. without clinician request) [14]. This suggests that in most institutions, cumulative antifungal susceptibility reports or antibiograms will not be available. Therefore, MICs are likely inferred from the most frequently isolated *Candida* species and regional/national susceptibility surveillance programmes [15], or based on established CLSI/EUCAST susceptibility breakpoints [6^▪▪^].

Clinical susceptibility breakpoints have been proposed for several triazole or echinocandin drug-species combinations by both CLSI and EUCAST [9,16,17]. These breakpoints, which link an MIC value measured *in vitro* with achievable drug concentrations *in vivo,* attempt to define MICs cut-offs associated with a higher probability of treatment failure. Several data sources are used to set breakpoints including MIC distributions, genetic markers of antifungal resistance, PK/PD data, usual antifungal dosing (EUCAST) and data from clinical trials describing patient outcome [6^▪▪^]. Less common yeast and moulds, however, lack sufficient clinical data to establish breakpoints. In these cases, an MIC value with an epidemiological cut-off value may be reported instead of a classical susceptibility designation, that is '*S, I or R*.’ The epidemiological cut-off value (ECV or ECOFF) encompasses the distribution of MICs for isolates without phenotypically or genotypically detected resistance mutations. Although not shown to be predictive of clinical outcome, an MIC result above the ECOFF may require altered dosing approaches or selection of an alternative treatment.

Recently, the EUCAST has changed definitions of susceptibility testing categories of ‘S, I, and R’ to clarify the relationship of drug dosing/exposure with the breakpoint designation. ‘S’ indicates *susceptible-to standard dosing regimen* when there is a high likelihood of therapeutic success using a standard dosing regimen; ‘I’ was changed from *intermediate* to *susceptible increased exposure* when there is a high likelihood of therapeutic success when drug exposure is increased by adjusting the dosing regimen or by virtue of high concentration at the site of infection. The revised ‘I’ category is analogous to *‘susceptible-dose-dependent (SDD)’* interpretative breakpoint used by the CLSI for fluconazole against *Candida* spp. when MICs are in the range of 4–32 μg/ml and can potentially be treated with higher doses (e.g. 12 mg/kg/day). ‘R’ is *resistant* when there is a high likelihood of clinical failure despite increased drug exposures. A fourth category of *area of technical uncertainty* (ATU) is assigned when the MIC is in an area where the MIC is not reproducible interpretable.

Individual MIC results are rarely available in the first week of empiric antifungal treatment. Therefore, it is often more practical to use and established breakpoint or ECOFF for the most likely pathogen(s) as the MIC target for initial PK/PD dosage calculations, even if an individual MIC value is found to be within the *susceptible* range or below ECOFF cut-offs [18]. For isolates with MICs just above the ECOFF without susceptibility breakpoints, some experts have recommended using a value of the ECOFF MIC plus 2 dilutions for PK/PD dosing calculations to minimize underdosing risk [18]. If the isolate MIC is several dilutions above the ECOFF or resistance breakpoint, MIC-guided therapy may not be feasible and alternative therapies should be considered.

A growing proportion of fungal infections are diagnosed based on molecular or antigen biomarkers such as β-D-glucan and galactomannan without culture diagnosis. The kinetics of β-D-glucan [19] and galactomannan [20,21] in response to antifungal treatment are predictive of clinical response to antifungal therapy and could possibly be analysed as an in vivo pharamcodynamic endpoint instead of the MIC using pharmacometric models [21].

The detection and interpretation of pharmacodynamic variability is less well standardized with antifungals versus antibacterials, making precision dosing with antifungals less certain. However, increasing antifungal resistance in yeast and moulds along with the anticipated approval of novel new antifungal therapies in the coming decade will place greater demand for clinically relevant and timely antifungal susceptibility testing results.

## PHARMACOKINETIC VARIABILITY AND THERAPEUTIC DRUG MONITORING

Pharmacokinetic variability is undoubtably an important contributor to treatment failure and toxicities associated with antifungal therapy [22]. The most common factors in critically ill patients that need to be considered are age, weight (obesity), altered volume of distribution associated with sepsis and/or hypoalbuminemia, altered renal function, extracorporeal circuits, including replacement therapy or extracorporeal membrane oxygenation (ECMO), and drug interactions. Bioavailability of oral triazole antifungal may be reduced in patients with chemotherapy-associated mucositis, graft-versus host disease involving the gut, diarrhoea and poor food intake, or altered gut perfusion [23]. Voriconazole is metabolized predominantly by CYP2C19 and to a lesser extent CY2C9 and CYP3A4. The drug exhibits nonlinear Michaelis-Menton pharmacokinetics in adults due to saturable clearance. Allelic variations in CYP2C19 contributes to high intra-individual pharmacokinetic variability, with poor metabolism genotypes associated with three-fold higher voriconazole exposures than patients who are homozygous extensive metabolizers [24].

Age is an important covariate affecting antifungal volume of distribution and clearance rates, with the greatest variability observed in paediatric populations. Clearance rates of fluconazole, voriconazole and posaconazole are more rapid in children and often resulting in subtherapeutic drug exposures unless higher or more frequent dosing is used [25]. Van der Elst *et al*. [26] reported that 40% of febrile neutropenic paediatric patients did not achieve recommended minimum therapeutic exposures (trough concentrations of < 11 mglL or AUC ≥ 400 mg•h/l) with fluconazole doses of 6 mg/kg/day administered in two divided doses. Voriconazole clearance is more rapid and unpredictable in children due to a three to five higher rate of CYP2C19 metabolism and enhanced activity of flavin-containing monooxygenase 3 [27,28]. Adequate concentrations of oral posaconazole may only be possible to achieve in paediatric patients who require weight-based dosing with a new fine-powder formulation [29].

Fluconazole and voriconazole require dosage adjustment based on total body weight an adjusted body weight, respectively [30]. Although conflicting data have been reported for itraconazole and posaconazole based on TDM studies, Wasmann *et al*. [31] found that higher doses of the intravenous posaconazole (400 mg twice daily loading, then 400 mg daily) were required to achieve similar exposures to standard 300 mg intravenous doses in patients weighing more than 140 kg. No dosage adjustment has been recommended for isavuconazole in obesity, but patients with low body mass index (< 18 g kg/m^2^) appear to be at a higher risk for supratherapeutic isavuconazole exposures with standard dosing [32].

All three echinocandins exhibit altered pharmacokinetics as weight increases above 65–75 kg [30]. In a recent empirical trial of micafungin for critically ill mechanically ventilated patients, micafungin 100 mg daily was found to be associated with at relatively lower (< 80%) probability of PK/PD target attainment for *C. albicans, C. glabrata* and *C. parapsilosis* in patients weighing more than 80 kg [33]. As a result, doses of 150 mg/day of caspofungin and micafungin are recommended, and loading and maintenance doses of anidulafungin should be increased by 25 and 50% in patients weighing more than 140 kg and more than 200 kg, respectively [34,35].

Significant intra-patient pharmacokinetic variability is observed with liposomal amphotericin B in both paediatrics [36] and adults [37]. This variability has been attributed to differences in saturable uptake of the liposome drug carrier into tissues or interactions with plasma proteins [37]. Limited pharmacokinetic data suggest that liposomal amphotericin B should not be administered based on total body weigh in obese patients, rather fixed doses of 300 or 500 mg should be considered in patients more than 100 kg [38].

### Therapeutic drug monitoring

Therapeutic drug monitoring (TDM) is the most direct method for detecting altered drug exposure in patients and has been recommended for patients receiving itraconazole, voriconazole, posaconazole suspension and flucytosine [23]. The most common approach for triazole antifungals is to sample trough concentrations once the patient reaches steady state typically 1-week into therapy, with empiric dosage adjustments to achieve recommended target serum trough levels (Table 1). However, changes in voriconazole pharmacokinetics occur more rapidly and initially sampling can be performed within the first 2–5 days. Conversely, the prolonged half-life of isavuconazole (mean 130 h) means that steady state may not be reached until 4 weeks into therapy.

**Table 1:** Pharmacokinetics/pharmacodynamics of antifungal agents

Antifungal class	Preclinical efficacy PK/PD target	Clinical PK/PD efficacy target	Clinical PK/PD toxicity target	Routine TDM recommended?	References
Amphotericin B deoxycholate	C_max_/MIC	Not established	Not established	No	[67–69]
Liposomal amphotericin B	AUC_0–24_/MIC (*Candida* spp.)AUC_0–24_/MIC (*Aspergillus* spp.)	Not established	Not established	No	[67,70]
Flucytosine	*f*T_>MIC_	> 20–45%T_>MIC_	C_max_> 100 mg/l(haematological and hepatic toxicity)	Yes;	[71]
Fluconazole	AUC_0–24_/MIC	AUC/MIC > 25–100; C_min_ 10–15 mg/l	Not established	No	[72,73]
Itraconazole	AUC_0–24_/MIC	C_min_ > 0.5 mg/l (prophylaxis);C_min_ >1 mg/l (treatment)	C_avg_: 17.1 mg/l (bioassay) 3–4 mg/l (HPLC)(gastrointestinal toxicity)	Yes	[74–77]
Voriconazole	AUC_0–24_/MIC	*f*AUC/MIC 25–50;C_min_ >1 mg/l or C_min_/MIC 2–5 mg/l	C_min_ > 5 mg/l(central nervous system toxicity)	Yes	[54,78–80]
Posaconazole	AUC_0–24_/MIC	*f*AUC/MIC 25–50;Prophylaxis C_min_ >0.5 mg/l or 0.7; treatment C_min_ > 1 mg/l	Not established	Yes	[81–83]
Isavuconazole	AUC_0–24_/MIC	*f*AUC/MIC 25--50	C_min_ > 5 mg/l(gastrointestinal toxicity)	No	[52,84]
Echinocandins	AUC_0–24_/MIC	AUC/MIC >3000	Not established	No	[85]

AUC_0–24_/MIC, 24 h area under the concentration-time curve to mean inhibitory concentration; C_avg_, average serum concentrations; C_max_/MIC, ratio of peak serum concentrations to MIC; C_min_, trough serum concentrations; C_min_/MIC ration of trough serum concentration to MIC; *f*T_>MIC_, duration of free (unbound drug concentration) remains above the MIC during the dosing interval.

The trough concentration (C_min_) is the most practical sample for estimating the AUC that drives efficacy of triazoles (Table 1). When analysed exposures fall outside proposed therapeutic ranges, triazole dosages are often adjusted empirically (i.e. increased or decreased by 50%) with repeat TDM follow-up. This approach to dose personalization is less precise and inefficient, especially for drugs such as voriconazole that exhibit extreme pharmacokinetic variability or altered clearance with increasing doses [39]. Pharmacometrics models for precise dosage calculation of voriconazole in adults and children has been developed and prospectively tested [27,40–42]. Collectively, these studies have shown improved precision and time to achieving target trough concentrations of 1–3 mg/l), although intensive TDM sampling (*n* = 4 samples per occasion per patient) was required to accurately model the nonlinear pharmacokinetic variability of voriconazole [40]. Although these models are useful for predicting optimal blood sampling times and interpreting TDM results, their use in patient care still requires regulatory approval in many countries and approaches to make them widely available and user-friendly are still in their infancy.

The importance of routine TDM with the newer posaconazole tablet formulations is still a matter of debate. In phase III clinical trials, 96% of patients achieved plasma C_min_ between 0.5 and 3.75 mg/l [43], although wide pharmacokinetic variability is still observed [44,45]. A higher rate of subtherapeutic exposures (Cmin < 0.7 mg/l during prophylaxis with posaconazole tablets has been reported in patients more than 90 kg, with severe diarrhoea or receiving protein pump inhibitors [46–48]. The presence of these risk factors, suspected noncompliance or breakthrough fungal infections would clearly favour documentation of posaconazole exposure by TDM. However, in patients without these risk factors receiving posaconazole for routine prophylaxis, the prevalence of ‘subtherapeutic’ posaconazole exposures may not be sufficiently high (> 10%) to justify the resources needed for routine TDM for posaconazole tablets [49]. Therefore, rational selective TDM based on specific clinical risk factors may be more appropriate [50].

The need for TDM during isavuconazole treatment is also unclear. Data from phase III trials did not identify specific dose--response relationships between the AUC, C_min_ and clinical response or all-cause mortality [51,52]. Clinical experience has also suggested most patients achieve putative therapeutic exposure (C_avg_ or C_min_> 1 mg/l) [53]. Patients who may benefit from TDM are patients with low body mass, suspected therapeutic failure, noncompliance or unexplained hepatotoxicity, or age less than 18 years [53].

While the importance of adequate exposures for clinical efficacy is generally accepted, the role of routine TDM to reduce toxicity risk is less clear. Trough concentrations of voriconazole more than 5.5 mg/l have been associated with increased rates of CNS adverse effects, but in the only prospective randomized trial of voriconazole TDM performed to date, the rate of CNS adverse effects was not reduced in patients randomized to TDM-guided dosage reduction versus the non TDM group [54]. The major benefit was that patients randomized to undergo routine TDM had voriconazole discontinued less frequently once adverse effects developed, which was ultimately associated with higher clinical success rates. Clinical experience suggests that subsequent serious CNS toxicities are uncommon in asymptomatic patients with supratherapeutic voriconazole concentrations [55]. Therefore, in the absence of toxicity, empiric dosage reductions, especially in patients with documented fungal infections, is probably not advisable based only on a single supratherapeutic concentrations and should probably only prompt closer follow-up and screening for potential explanations such as drug interactions.

## DRUG-DRUG INTERACTIONS

Pharmacokinetic drug-drug interactions are highly prevalent among patients receiving mould-active triazoles that are potent inhibitors of cytochrome P450 3A4, occurring in between 75 and 88% of hospital admissions [56]. Up to 75% of drug interactions can be classified as severe depending on the mould-active triazole prescribed [56]. The clinical severity of drug-drug interactions with mould-active triazoles is generally driven by two factors: the magnitude of the interaction caused by the perpetrator drug; and the therapeutic index of the victim drug. When triazoles are the victim drug, coadministration of perpetrator drugs that affect oral bioavailability (e.g. omeprazole with posaconazole suspension) or accelerate metabolism and clearance of the triazole (e.g. CYP3A4 inducer rifampin with any triazole) and can lead to insufficient drug exposures and a high risk of treatment failure. Avoidance of these combinations, or in the case of weaker perpetrators, TDM documentation of antifungal serum concentrations with careful clinical monitoring and dosage adjustment if needed, is essential when starting and stopping antifungal therapy.

The most common clinical scenario for drug interactions with triazoles involves mould-active triazoles acting as a potent perpetrator to inhibit CYP3A4-emdiated metabolism of a victim drug. When the therapeutic index of the victim drug is narrow, as is the case of chemotherapy for haematological malignancies or immunosuppressive therapy administered after transplantation, then coadministration of mould-active triazoles should be avoided or preemptive dosage reduction of the victim drug is essential.

The problem of drug interactions with mould-active triazoles has become even more important in the last 5 years with exploding number of small-molecule kinase inhibitors (SMKIs) introduced into clinical practice as targeted therapy for lymphoid and myeloid malignancies. The majority of these SMKIs metabolized through CYP3A4/5 and have a narrow therapeutic range and potentially severe dose-dependent side effects including prolongation of the QTc interval [57^▪▪^]. Drug interactions with these agents differs from traditional chemotherapy, however, in that these agents are administered orally primarily in the outpatient setting for prolonged periods. Unfortunately, limited medical guidance for managing SMKI- triazole drug interactions is available, and clinicians often are forced to consider forgoing antifungal prophylaxis or treatment with triazoles, use alternative intravenous-only prophylaxis, or continue both drug at full-dose strength and carefully monitor for toxicities [58].

One of the most frequently prescribed SMKIs, venetoclax, an inhibitor of B-cell lymphoma-2 (BCL-2) protein that regulates cellular apoptosis. Venetoclax is used for the treatment of chronic lymphocytic leukaemia, small lymphocytic lymphoma and acute myelogenous leukaemia in combination with hypomethylating agents (azacitidine, decitabine). When administered in combination with posaconazole, the C_max_ and AUC of venetoclax is increased 7.1 and 8.8-fold, respectively [59] resulting in prolonged thrombocytopenia and neutropenia. As a result, it is recommended that the dose of venetoclax be reduced by at least 75% with strong CYP3A4 inhibitors (itraconazole, voriconazole, posaconazole) and at least by 50% is moderate CYP3A4 inhibitors (fluconazole, isavuconazole). However, the duration of neutropenia and thrombocytopenia with venetoclax was not necessarily predicted by the strength of the inhibitors, as patients receiving isavuconazole exhibited significantly longer times to neutrophil and absolute neutrophil count recovery versus voriconazole and posaconazole [60,61,62^▪^]. Similar dosage recommendations have been proposed or are probably applicable for SMKIs that target PI3Kδ (idelalisib), Bruton-tyrosine kinase (ibrutinib), Janus-kinase-1 and -2 (ruxolitinib), isocitrate dehydrogenase1 or 2 (ivosidenib, enasidenib), and Bcr-Abl fusion gene tyrosine kinase inhibitors (imatinib, ponatinib, nilotinib, bosutinib)- as discussed in more detail in recent reviews [57^▪▪^,^63^,^64^].

Inhibitors of FMS-like tyrosine kinase 3 ligand (FLT-3) such as midostaurin, sorafenib and gilteritinib, pose a major dilemma as these SMKIs are used either as part of an induction chemotherapy regimen or as salvage/rescue therapy for patients with acute myelogenous leukaemia, a particularly high-risk patient group for invasive mould disease [65]. Coadministration of midostaurin with potent CYP3A4-inhibitors (voriconazole, posaconazole) was associated with an accelerated time to Grade III/IV toxicities, but dosage-reduction of midostaurin was associated with significantly higher risk of AML relapse or death [66].

Further complicating matters, the pharmacology of many SMKIs is complex. Similar, to triazoles, these targeted therapies exhibit high degrees of intra-individual pharmacokinetic variability and several drugs have active metabolites proposed to contribute to antitumor activity. Therefore, it is unclear whether simple fixed dosing adjustments similar to what is proposed for venetoclax will be possible or effective.

TDM in combination with direct measurement of pharmacodynamic biomarkers of tumour response has been proposed as a method to improve precision dosing of SMKIs and manage drug interactions, but pharmacometric models required to support this strategy are currently in early stages of development and validation [63]. As a result, the management of antifungal drug interactions, with targeted SMKIs for haematological malignancies remains largely empirical and imprecise.

## CONCLUSION

Pharmacokinetic and pharmacodynamic variability is commonly encountered in patients with invasive fungal disease, making dosage selection less certain. Although susceptibility testing for fungi has continued to progress over the last two decades, MIC data are rarely in hand at the time of initial regimen selection placing a greater importance on rapid and accurate pathogen identification, knowledge of local epidemiology and resistance patterns. Pharmacokinetic variability is best detected by TDM but is currently only employed in a crude fashion for triazole antifungals for dosage adjustment or evaluation of treatment failure or unexpected toxicities. Ultimately, precision dosing of antifungal therapy will require greater development of user-friendly pharmacometric models that can be individualized through TDM to identify treatment regimens with the greatest probability of treatment success without toxicity. Similar approaches will be needed for SMKIs when used in combination with triazole antifungals to ensure the safety and efficacy of these lifesaving targeted chemotherapies.

## Acknowledgements

*None.*

### Financial support and sponsorship

*None.*

### Conflicts of interest

*There are no conflicts of interest.*

## REFERENCES AND RECOMMENDED READING

Papers of particular interest, published within the annual period of review, have been highlighted as:

▪ of special interest
▪▪ of outstanding interest
